# Deep learning-enabled hyperspectral imaging for high-accuracy non-destructive quantification of nutritional components in multi-variety apples

**DOI:** 10.3389/fpls.2025.1634785

**Published:** 2025-09-18

**Authors:** Hanhan Zhai, Pan Xie, Xin Xie, Shuai Shuai Sha

**Affiliations:** ^1^ School of Advanced Agricultural Sciences, Kashi University, Kashi, China; ^2^ Agricultural Science Institute, 3rd Division of Xinjiang Production and Construction Corps, Tumxuk, China

**Keywords:** hyperspectral imaging, deep learning, non-destructive detection, apple quality parameters, multi-attribute quantification

## Abstract

Conventional methods for quantifying soluble solids content (SSC), vitamin C (VC), and soluble protein (SP) levels in apples are destructive and unsuitable for large-scale postharvest quality monitoring. This study aimed to develop a convolutional neural network-bidirectional gated recurrent unit-attention (CNN-BiGRU-Attention) model based on hyperspectral imaging (HSI) to achieve high-precision non-destructive quantification of VC, SSC, and SP in apples. The model was established using six apple varieties from diverse geographical origins, leveraging hyperspectral data spanning 400–1000 nm with 512 spectral bands. The model framework demonstrated superior performance with raw hyperspectral cube inputs. Optimal predictions for VC and SSC were achieved using full-spectrum modeling (test set: R²_VC_=0.891, R²_SSC_=0.807, RPD _VC_=3.117, RPD _SSC_=2.337). For SP quantification, feature wavelength selection (403, 430, 551, 617, and 846 nm) via successive projections algorithm (SPA) yielded R²=0.848, RPD=2.642, which aligned with the N-H/C-H vibrational overtones and aromatic amino acid absorption bands. Cross-year validation of 2024 hyperspectral dataset confirmed the robustness of the model, with R^2^ values of 0.829, 0.779, and 0.835 (RPD>2.000) for VC, SSC, and SP, respectively. Taken together, this study resolves high-dimensional data redundancy through hybrid architectures and offers a deployable solution for multi-variety fruit quality monitoring.

## 1 Introduction

Apple (*Malus domestica*), a globally cultivated pome fruit, is highly valued for its rich nutritional composition and distinctive flavor profile, which makes it a crucial agricultural commodity (Hu Z. et al., 2025). The nutritional quality and sensory characteristics of apples are primarily determined by their soluble solids content (SSC), ascorbic acid (vitamin C, VC), and soluble protein (SP) levels (Hu et al., 2024). Conventional methods for quantifying these parameters include refractometry for SSC (Vega-Castellote et al., 2024), high-performance liquid chromatography (HPLC) or 2,6-dichlorophenolindophenol (DCPIP) titration for VC (Hao et al., 2025), and the Bradford colorimetric assay for SP (Chen et al., 2025). However, these methods are destructive, labor-intensive, and unsuitable for large-scale, continuous postharvest quality monitoring.

Hyperspectral imaging (HSI) is an intelligent non-destructive detection method that has emerged as a promising technique in recent years, with advantages of rapid, cost-effective, and non-invasive analysis (Huang et al., 2025). The HSI technique integrates spatial-spectral signatures with chemometric modeling to facilitate simultaneous prediction of multiple quality metrics, and it has been successfully applied to apples (Razavi et al., 2025b), bananas (Sripaurya et al., 2021), citrus (Jiang et al., 2025), peaches (Chen et al., 2024), and cherries (Zheng et al., 2025). Despite these advantages, Bai et al. demonstrated that HSI models suffer from generalization decay when applied across apple varieties, geographical origins, or growing seasons due to environmental heterogeneity (Bai et al., 2019).

Traditional HSI modeling relies on partial least squares regression (PLSR) and support vector machines (SVM) (Zeng et al., 2024; Günaydın et al., 2025; Razavi et al., 2025a), which require extensive spectral preprocessing and manual feature selection. These methods lack adaptive learning capabilities and are inadequate for high-dimensional spectral-spatial data. Deep learning (DL) architectures, particularly convolutional neural networks (CNNs), have revolutionized chemometrics, as they enable end-to-end extraction of hierarchical non-linear features from raw hyperspectral cubes and eliminate dependency on manual preprocessing (Mansuri et al., 2022; Yuan et al., 2025). Comparative studies have confirmed that CNNs are superior to linear methods in complex spectral-spatial decoding tasks (Kaur et al., 2024; Sun et al., 2025; Wang et al., 2025).

However, CNNs primarily capture local features and are less effective at modeling the sequential nature of spectral data, which often exhibit long-range dependencies along the wavelength axis. To address this, we further introduce Bidirectional Gated Recurrent Units (BiGRUs) to enhance the model’s ability to learn contextual spectral information in both forward and backward directions (Feng et al., 2023). BiGRUs are particularly suitable for hyperspectral applications because they effectively capture temporal relationships across spectral bands while maintaining a lightweight structure. Compared with traditional recurrent networks like LSTMs, BiGRUs have fewer parameters, faster convergence, and are more computationally efficient, which makes them advantageous for applications with limited sample sizes and real-time processing requirements. These characteristics make BiGRUs not just a convenient choice, but a functionally appropriate one for modeling spectral sequences in agricultural products. For instance, Jiao et al. reported a 97.54% accuracy in maize moisture prediction using a temporal convolutional network-BiGRU (TCN-BiGRU) hybrid model, which outperformed standalone CNNs (Yang et al., 2025). Li et al. achieved a 99.21% classification accuracy for *Panax quinquefolius* origin tracing (Li et al., 2025), and Hu et al. improved rice yield prediction by combining CNNs with spectral attention (Hu T. et al., 2025). Although existing studies predominantly focused on single-origin or single-variety predictions (Li et al., 2018; Fan et al., 2019; Guo et al., 2023), integrated frameworks that address cultivar, geographical, and seasonal variability remain underexplored. Thus, robust, universally applicable models need to be developed to advance the HSI technology in practical agricultural settings.

This study leveraged HSI to acquire hyperspectral data from six apple varieties cultivated across diverse geographical regions in 2023. We developed CNN, CNN-BiGRU, and CNN-BiGRU-Attention models to predict VC, SSC, and SP levels using the successive projections algorithm (SPA) for feature wavelength selection. External validation using a 2024 dataset confirmed the robustness of the model. The developed framework provides a theoretical and technical foundation for rapid, nondestructive apple quality assessment to address critical challenges in multi-variety and cross-regional applications.

## 2 Materials and methods

### 2.1 Research procedures

**Figure 1:**
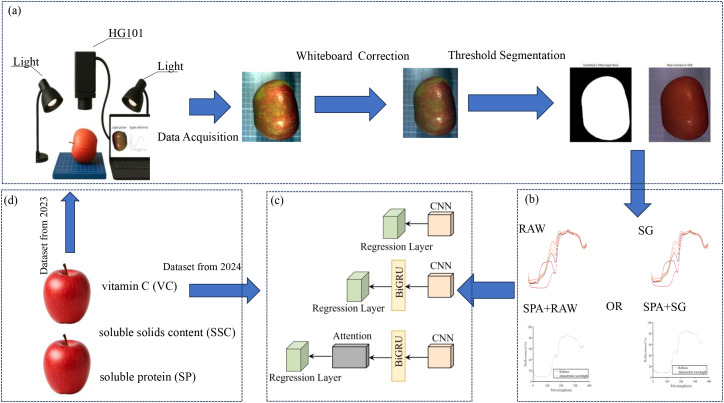
Flowchart of the experimental process. **(a)**: Diagram of the data acquisition setup and hyperspectral image processing, **(b)**: Data preprocessing, **(c)**: Deep learning model, **(d)**: Measurement indicators and data sources.

**Figure 1** illustrates the schematic workflow of the proposed deep learning-based apple quality prediction system, which comprises four core phases. First, in the data acquisition stage, hyperspectral images of apples are collected using a hyperspectral imaging system, followed by white reference correction. Regions of interest (ROIs) are extracted through a series of image processing steps, including image enhancement, binary segmentation, connected component analysis, contour extraction, B-spline fitting, and smoothing, to ensure accurate retrieval of spectral reflectance. Second, in the feature selection phase, Savitzky–Golay (SG) preprocessing is combined with the Successive Projections Algorithm (SPA) to extract key spectral bands that are most informative for quality prediction. Third, during model construction, three deep learning models—CNN, CNN-BiGRU, and CNN-BiGRU-Attention—are developed to predict VC SSC and SP based on spectral reflectance. Finally, in the model training and validation phase, data collected in 2023 is used for model training, while data from 2024 serves as an independent test set to evaluate model robustness and generalization performance.

### 2.2 Experimental materials

The apple sample library constructed in this study encompasses three major Chinese production regions (Xinjiang; North China; Jiaodong Peninsula) and comprises 144 samples from six representative cultivars to integrate dual heterogeneity in geographical origin and genetic resources. The spatiotemporally stratified design included the following three geographical indication cultivars: Xinjiang Aksu Red Fuji (AKS) (41.17°N, 80.26°E; *n* = 16), Hebei Shunping Red Fuji (SPFS) (38.85°N, 114.18°E; *n* = 16), and Shandong Yantai Red Fuji (YTFS) (37.47°N, 121.45°E; *n* = 16), in addition to three non-GI cultivars—Cherry Apple (YT), *n* = 16; Ralls Janet (GG), *n* = 16; and Huaniu Apple (HN), *n* = 16. The samples were collected in the 2023–2024 growing seasons, with 96 baseline samples collected in 2023 and 48 additional validation samples in 2024. This framework effectively balanced cultivar genetic backgrounds, regional climatic characteristics, and harvest timing variables to provide robust data support for generalizable spectral modeling: the 2023 dataset was partitioned into training and testing sets (7:3 ratio), and the 2024 dataset served as an external validation set.

### 2.3 Physiological parameter determination

#### 2.3.1 VC quantification

A 1.0 g apple sample was homogenized in a mortar with 5 mL of 2% oxalic acid solution. The homogenate was quantitatively transferred to a 10 mL volumetric flask, diluted to the mark volume with oxalic acid solution, and then filtered. A 10 mL aliquot of ascorbic acid standard solution (0.1 mg/mL) was titrated with standardized 2,6- DCPIP solution until it reached a persistent rose-red endpoint (15-second stability), with dye consumption recorded for titrant standardization. Thereafter, 5 mL of the sample filtrate was similarly titrated, and VC content was calculated based on dye consumption (Hao et al., 2025).

#### 2.3.2 SSC measurement

Following NY/T 2637-2014 (Fruit and Vegetable Products - Determination of Soluble Solids by Refractometry), three 2-mm-thick flesh slices were stacked (total thickness: 3 mm) and juiced using a hydraulic press (5 kN). The filtered juice (80-mesh sieve) was analyzed in triplicate using a PR-101α digital refractometer (Atago Co., Japan), with mean values from triplicate measurements (both sides of each slice) recorded as final SSC values (Vega-Castellote et al., 2024).

#### 2.3.3 SP assay

A 0.5 g sample was homogenized with 2.0 mL distilled water and centrifuged (4,000 ×*g*, 10 min). The supernatant was diluted (0.20 mL supernatant + 0.80 mL water) and allowed to react with 5.00 mL Coomassie Brilliant Blue G-250 staining solution for 2 min. The absorbance at 595 nm was measured using a UV-1800 spectrophotometer (Shimadzu, China), with SP concentration determined via a bovine serum albumin standard curve (Chen et al., 2025).

### 2.4 Hyperspectral imaging acquisition and spectral data processing

#### 2.4.1 Hyperspectral imaging system

Hyperspectral data acquisition was conducted using a push-broom hyperspectral imaging system (HG101, Nakagawa Photonics, China), which covers a spectral range of 395–1008 nm with a spectral resolution of 2.8 nm and acquires 360 contiguous bands. The imaging system was equipped with dual light source irradiation modules—two 150 W fiber-optic halogen lamps were symmetrically positioned on both sides of the sample stage at a height of 250 mm to form a 45° irradiation angle with the sample plane to ensure over 95% surface illumination uniformity. A 30-minute preheating procedure was strictly implemented prior to the experiments to ensure that the detector reached thermal equilibrium within the 395–1008 nm wavelength range and to minimize dark current noise interference. To eliminate external factors and instrument effects, raw hyperspectral images were corrected using a white reference panel before spectral extraction.

#### 2.4.2 Spectral data extraction and preprocessing

As shown in (**Figure 1a**), after white reference correction, individual apple regions were segmented using a thresholding-based method to obtain complete hyperspectral images. Spectral reflectance data across 360 bands were then extracted, and the average reflectance within the selected ROIs was calculated as the original spectral data for each sample, yielding corresponding wavelength-reflectance curves. To improve the accuracy of region extraction, a method was adopted that integrates spectral difference information with B-spline-based contour modeling for precise target segmentation and fitting in hyperspectral images. This approach offers several advantages: it enhances target boundaries by leveraging spectral contrast; it automatically selects the largest connected component, ensuring high robustness; it improves the geometric continuity and smoothness of contours using B-spline fitting; and it provides clear and intuitive visual outputs for further analysis. This preprocessing workflow enables accurate and interpretable target extraction, providing high-quality input data for subsequent feature selection and model construction.

#### 2.4.3 Spectral data preprocessing

Spectral preprocessing, a critical data optimization method in chemometrics, effectively removes interference signals that are unrelated to target variables while enhancing valid spectral features (Leoni et al., 2024; E et al., 2025). In this study, SG smoothing was applied as the sole preprocessing method to suppress random spectral noise and preserve local signal trends. Unlike traditional chemometric models that heavily rely on spectral correction techniques such as Standard Normal Variate and Multiplicative Scatter Correction, the proposed deep learning architectures (CNN, CNN-BiGRU, and CNN-BiGRU-Attention) incorporate internal normalization mechanisms (e.g., batch normalization and weight adjustment through backpropagation). Therefore, additional scatter correction methods were not applied to avoid redundant normalization and potential information loss. The SG convolution smoothing method, based on the principle of locally weighted least squares, suppresses high-frequency noise by constructing polynomial fitting models within sliding temporal windows. This method adaptively eliminates random noise interference in spectral data through parameterized adjustments of window width and polynomial order while preserving original signal waveform characteristics (Zhang et al., 2024).

#### 2.4.4 Feature extraction

In spectral detection, the high dimensionality and complexity of acquired data often lead to information redundancy or model overfitting (Wang et al., 2020). To address this issue, the SPA was introduced for feature wavelength selection. SPA is an efficient feature wavelength screening method that helps to extract informative and non-redundant spectral bands from high-dimensional data. The algorithm employs an iterative strategy, i.e., initial selection of wavelength with the highest variance in the spectral matrix to prioritize the most informative regions, followed by orthogonal projection calculations of the remaining wavelengths in the selected band space to identify wavelengths with maximum projection values for inclusion in the feature set (Yu et al., 2025). In this study, SPA was chosen primarily based on its proven effectiveness in similar hyperspectral modeling tasks, as well as its computational efficiency and ability to produce compact, interpretable feature subsets suitable for deep learning model input (Zhao et al., 2022; Vallese et al., 2024).

Specifically, the SPA procedure in this study was executed in three phases to ensure stability and reproducibility. In Phase 1, all spectral variables were standardized to zero mean and unit variance. Each variable was used as an initial projection point, and candidate subsets were generated via orthogonal projections, with the number of iterations ranging from a minimum of 5 to a maximum of 360 wavelengths. In Phase 2, all subsets were evaluated using the Prediction Residual Error Sum of Squares (PRESS) criterion based on an independent validation set. The optimal subset was determined by the combination that yielded the lowest PRESS value. In Phase 3, variables were ranked according to a relevance index derived from regression coefficients and variable standard deviations, and a final subset was selected using an F-test (α = 0.25) to ensure that additional variables did not significantly increase prediction error.

### 2.5 Model construction

This study employed the CNN, CNN+BiGRU, and CNN+BiGRU+Attention models to predict apple VC, SSC, and SP levels. Raw spectral and preprocessed data were used as input parameters to establish quantitative prediction models. Full-spectrum and feature-selected bands served as independent variables, whereas VC, SSC, and SP served as dependent variables.

**Figure 2:**
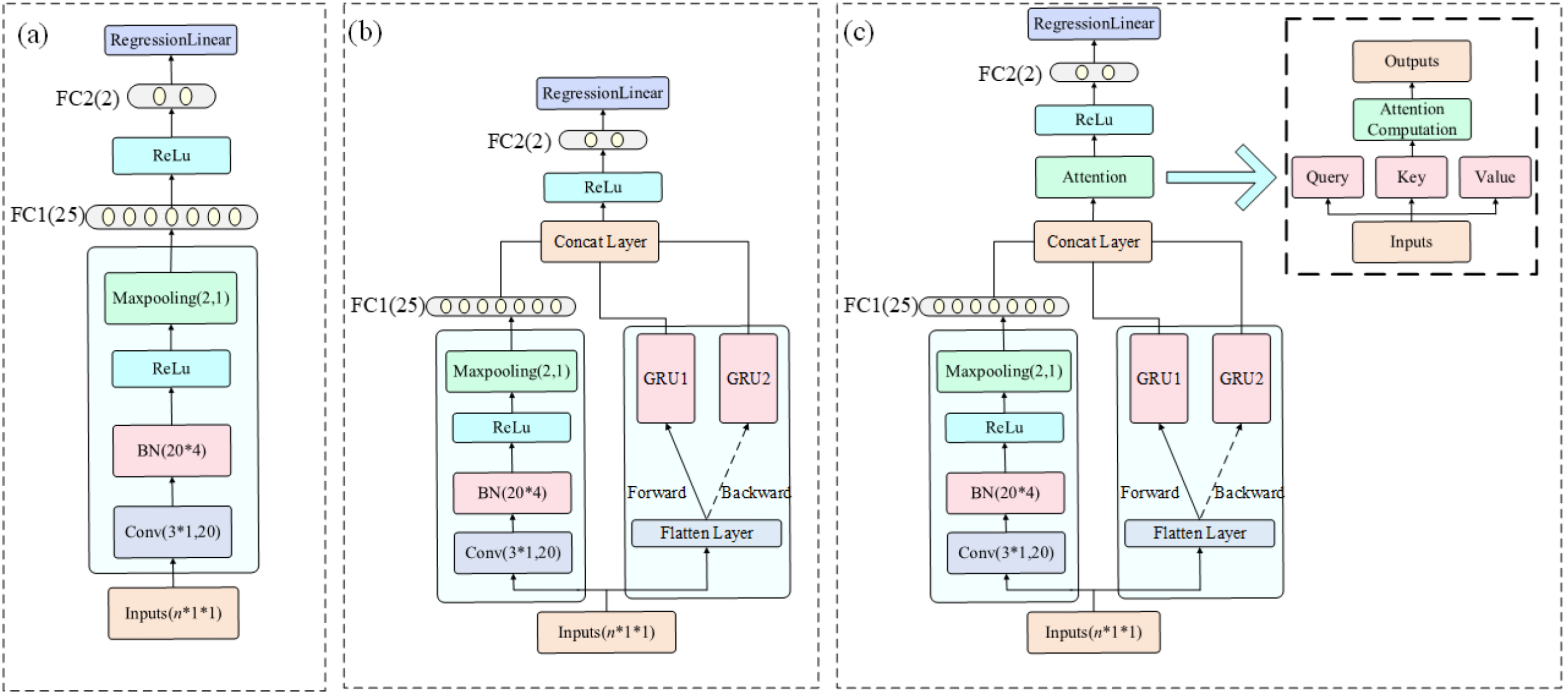
Structural diagrams of the three deep learning models. The input format is *n × 1 × 1*, where *n* denotes the number of input spectral bands, which varies depending on whether full-spectrum data or SPA-selected features are used: **(a)** CNN, **(b)** CNN+BiGRU, and **(c)** CNN+BiGRU+Attention.

The CNN model effectively identifies local spatial-spectral features through convolutional and pooling operations. The input spectral vector is reshaped into a pseudo-image of size [n × 1 × 1], where n is the number of selected wavelengths. The network consists of two convolutional layers with kernel sizes of [3×1] and filter sizes of 8 and 32, each followed by batch normalization, ReLU activation, and [2×1] max pooling. A dropout layer with a rate of 0.4 is applied for regularization. The output is then passed through two fully connected layers (with 32 and 1 neurons) and a regression layer for final prediction. This model enables autonomous feature learning and captures local non-linear structures in the spectral domain, as shown in (**Figure 2a**).

The CNN-BiGRU model enhances the CNN by incorporating a BiGRU to capture global dependencies and sequential patterns in the spectral data. The architecture includes two parallel branches: a CNN stream with a convolutional layer (16 filters, [3×1] kernel), batch normalization, ReLU, [3×3] max pooling, flattening, and a fully connected layer with 25 neurons; and a BiGRU stream with two GRU layers (35 hidden units each), processing both the original and reversed sequences via a flip layer. Outputs from both branches are concatenated and passed to a regression layer. The bidirectional design enables simultaneous analysis of short-to-long and long-to-short wavelength relationships, revealing interactions across absorption peaks and baseline drift, as illustrated in (**Figure 2b**).

To further strengthen spectral feature representation, the CNN-BiGRU-Attention model incorporates a self-attention mechanism following the feature concatenation step. A single-head self-attention layer (key and query dimensions = 50) is employed using dot-product attention to dynamically assign weights to spectral components. This integration of 1D convolution, bidirectional temporal modeling, and adaptive attention enables the network to focus on the most informative spectral regions while suppressing noise, as shown in (**Figure 2c**).

All models were trained using the Adam optimizer. The CNN model was trained for 500 epochs with an initial learning rate of 0.001 and an L2 regularization factor of 0.01. For the more complex CNN-BiGRU and CNN-BiGRU-Attention models, training was extended to 1000 epochs with a higher initial learning rate of 0.01. A learning rate decay strategy was applied, reducing the rate by a factor of 0.1 after 600–800 epochs.

These training configurations were carefully tailored to each model’s structural complexity and convergence behavior to achieve optimal performance and generalization. For instance, the CNN model, with its relatively shallow architecture and fewer parameters, converged effectively under a moderate learning rate (0.001) and shorter training duration (500 epochs). In contrast, the CNN-BiGRU and CNN-BiGRU-Attention models, which introduce sequential learning and attention mechanisms, possess deeper structures and higher parameter counts. These models required a larger initial learning rate (0.01) and longer training (1000 epochs) to fully converge. A learning rate decay was introduced after epoch 600 to stabilize optimization and prevent overshooting in the fine-tuning phase. These settings were determined through preliminary trials and empirical adjustments, balancing training speed, overfitting risk, and predictive accuracy.

### 2.6 Model evaluation metrics

Through the screening of spectral regression models for quantitative prediction of apple VC, SSC, and SP, a multidimensional evaluation framework was established to balance numerical accuracy and model robustness (Cui et al., 2025). R² measures the proportion of variance in the reference data explained by the model. A value closer to 1 indicates better fit. The equation is as follows, as shown in (Equation 1):


(1) $${\text{R}}^{2}=1-\frac{\sum_{i=1}^{n}{\left(y_{i}-{\hat{y}}_{i}\right)}^{2}}{\sum_{i=1}^{n}{\left(y_{i}-\bar{y}\right)}^{2}}$$

where $y_{i}$ is the actual measured sample value, ${\overset{⌢}{y}}_{i}$ is the predicted sample value, $\bar{y}$ is the mean actual measured sample value, and n is the number of samples in the set.

The root mean squared error (RMSE) evaluates the average magnitude of prediction errors. Lower RMSE indicates higher accuracy. It is mathematically expressed as follows, as shown in (Equation 2):


(2) $$RMSE=\sqrt{\frac{1}{n}\sum_{i=1}^{n}{\left(y_{i}-{\hat{y}}_{i}\right)}^{2}}$$

where $y_{i}$ is the actual measured sample value, ${\overset{⌢}{y}}_{i}$ is the predicted sample value, and n is the number of samples in the set.

RPD is the ratio of the standard deviation of reference values to the RMSE. It reflects the robustness and generalization of the model. Generally, RPD > 2.0 indicates good predictive performance, while RPD ≤ 1.4 implies weak prediction (Zhang et al., 2025). RPD is expressed as shown in (Equation 3):


(3) $$RPD=\frac{SD}{RMSE}$$

where SD denotes the standard deviation of measured values.

### 2.7 Statistical analysis

One-way ANOVA was conducted using SPSS software (v23.0; SPSS Inc., Chicago, USA), and significant differences between treatments were assessed with Duncan’s multiple range test (*p*< 0.05).

## 3 Results and discussion

### 3.1 Analysis

**Figure 3:**
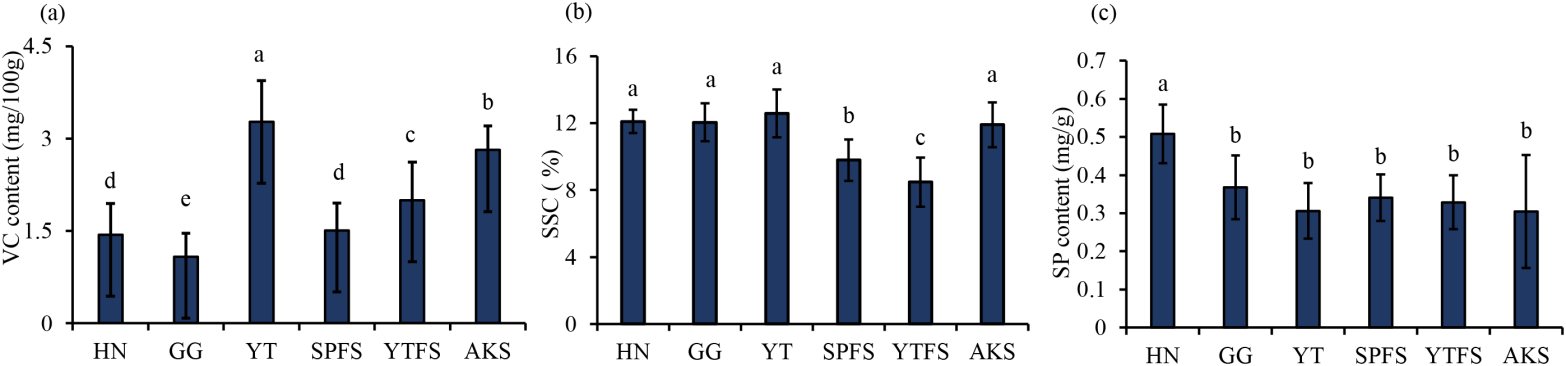
Internal quality parameters of apple samples: **(a)** VC; **(b)** SSC; **(c)** SP. SSC, soluble solids content; VC, ascorbic acid (vitamin C); SP, soluble protein. Significance analysis: Different letters (a, b, c, d, e) indicate statistically significant differences between groups (p < 0.05).

A systematic analysis of VC, SSC, and SP levels was conducted across 96 apple samples collected in 2023 (**Figure 3**). The results revealed significant varietal differences: YT had the highest VC content (3.27 ± 0.67 mg/100 g, *p*< 0.05), whereas GG had the lowest VC levels (1.08 ± 0.38 mg/100 g). No significant differences in SSC levels were observed among most of the cultivars (*p* > 0.05), except for YTHFS and SPHFS. Notably, HN apples demonstrated 27–40% higher SP content (0.51 ± 0.08 mg/g, *p*< 0.05) than the other varieties.

Geographical analysis indicated that AKS had significantly higher levels of both VC (2.82 ± 0.39 mg/100 g) and SSC (11.92 ± 1.34%) compared to YTHFS (VC: 2.00 ± 0.62 mg/100 g; SSC: 8.48 ± 1.48%) and SPHFS (VC: 1.51 ± 0.44 mg/100 g; SSC: 9.80 ± 1.23%) (*p*< 0.05). This could be likely attributable to distinct ecological factors such as diurnal temperature variation and prolonged annual sunshine duration in Aksu (Bai et al., 2019). No significant SP differences were observed among the Red Fuji apples.

**Figure 4:**
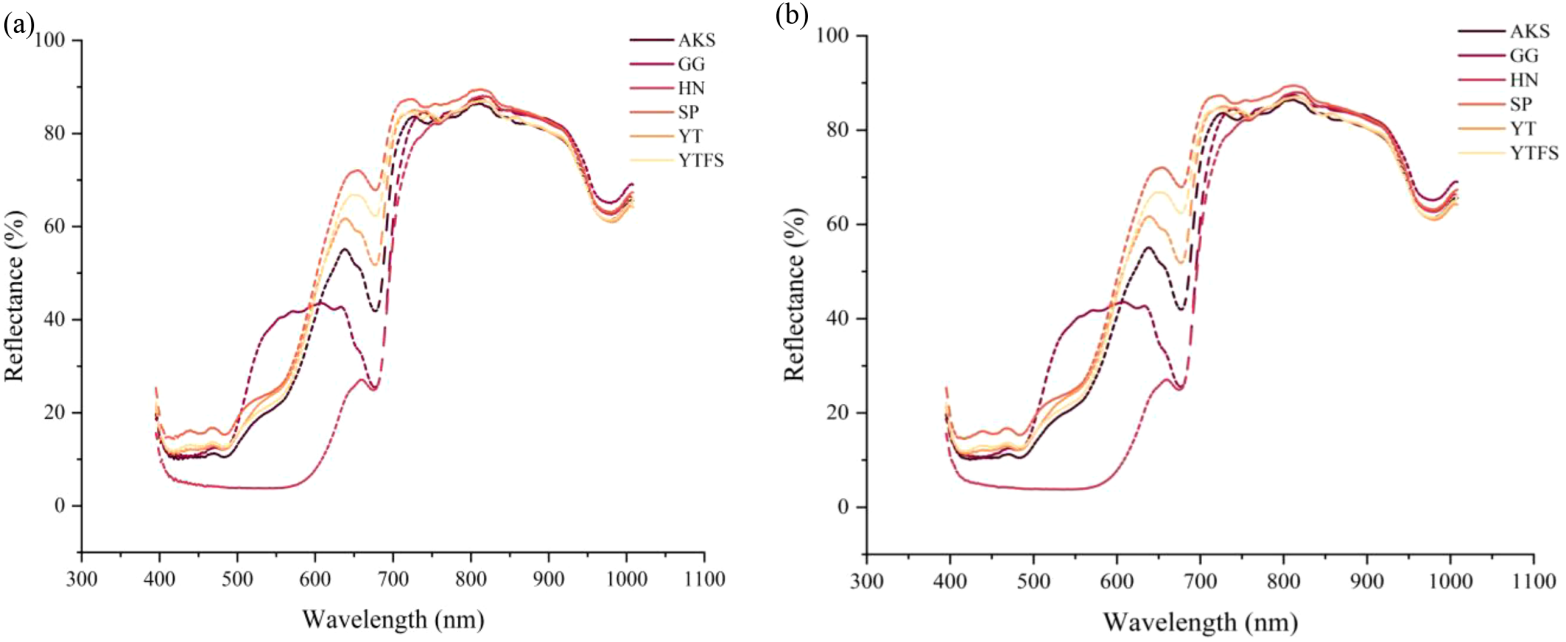
Spectral reflectance and preprocessing reflectance. **(a)** The raw reflectance **(b)** SG smoothing preprocessing.

The broad distribution of chemical indices across four cultivars and three agroecological zones established essential chemical gradients and biological diversity for hyperspectral modeling, with VC levels at 1.08–3.27 mg/100 g, SSC at 8.48–11.92%, and SP levels at 0.32–0.51 mg/g.

(**Figure 4a**) presents the visible-near infrared (Vis-NIR) spectral reflectance characteristics of six apple cultivars from different geographical origins, with the horizontal axis representing the wavelength range (395–1008 nm) and vertical axis indicating the reflectance intensity. Spectral analysis revealed that HN apples exhibited significantly lower reflectance in the 400–600 nm visible range than the other cultivars, probably due to their deeper epidermal pigment deposition. Although the reflectance values of other cultivars showed minor fluctuations, their overall spectral curves maintained similar morphological trends, which is consistent with the findings reported by Wang et al (Wang et al., 2024).

The observed spectral variations among different cultivars were closely associated with their intrinsic physiological properties. Distinct reflectance differences in the 400–700 nm range were particularly noticeable across geographical origins and cultivars, probably due to the variations in pigment composition, especially anthocyanin and chlorophyll contents. As demonstrated by Mark et al (Merzlyak et al., 2003), anthocyanins exhibit maximum sensitivity in the 400–675 nm spectral region, with elevated concentrations corresponding to reduced reflectance; our experimental data corroborated this finding. The reflectance variation at approximately 680 nm primarily originated from chlorophyll content differences, whereas the spectral fluctuation at 970 nm was associated with moisture variations in apple tissues, which correspond to the third overtone absorption band of O-H stretching vibrations.

(**Figure 4b**) displays the SG-smoothed spectra after pretreatment and show significantly enhanced spectral smoothness compared to the original data. This observation aligns with the results of Hu et al (Hu Z. et al., 2025).

### 3.2 Feature wavelength extraction

The narrow intervals between adjacent bands in raw spectral data typically cause substantial data redundancy (Zhu et al., 2025). To address this issue, the SPA was employed for feature wavelength extraction. As summarized in **Table 1**, the selected feature wavelengths spanned the entire wave number range. Specifically, the SPA identified 5, 33, and 5 spectral variables as characteristic wavelengths for VC, SSC, and SP parameters in raw data, but 13, 11, and 5 variables were selected for the corresponding parameters in pretreated data. This feature extraction approach not only retains critical spectral information but also effectively mitigates inter-variable correlations.

**Table 1:** Extraction results of feature-selected parameters.

Preprocessing	Parameters	Characteristic wavelengths (nm)
RAW	VC	409, 577, 679, 700, 757
SG	VC	395, 398, 409, 423, 481, 506, 585, 617, 651, 700, 751, 777, 1008
RAW	SSC	397, 398, 400, 403, 405, 406, 411, 412, 416, 419, 422, 430, 468, 504, 519, 585, 615, 679, 690, 702, 719, 793, 846, 860, 874, 928, 949, 965, 974, 994, 1001, 1003, 1008
SG	SSC	395, 400, 423, 539, 591, 632, 679, 828, 846, 925, 1008
RAW	SP	403, 430, 551, 617, 846
SG	SP	400, 641, 662, 974, 994

SSC, soluble solids content; VC, ascorbic acid (vitamin C); SP, soluble protein; SG, Savitzky-Golay.

Based on previous spectral studies, many of the selected wavelengths (e.g., 679, 700, 757 nm) correspond to known absorption features of O–H and C–H groups (Magwaza et al., 2012), which are associated with the presence of sugars, organic acids, and polyphenols in apples (Shao and He, 2007). In particular, regions near 700–950 nm have been widely reported to correlate with SSC and vitamin C content, as they reflect the third overtone of O–H stretching and water absorption characteristics (Abbas et al., 2017). Many of the selected wavelengths for SP prediction (e.g., 403, 430, 617, 846, 974, and 994 nm) correspond to characteristic absorption bands of O–H, C–H, and N–H bonds, which are commonly found in soluble proteins and their constituent amino acids (Zhang et al., 2019; Fu et al., 2022). The visible bands (400–430 nm) may reflect the interaction of proteins with chromophores or pigments (Fan et al., 2020; Zhang et al., 2023). The red region (550–660 nm) has been associated with amino acid signatures, while the near-infrared bands around 846 nm and especially 974 and 994 nm are related to overtone absorptions of O–H and N–H stretching vibrations, which are typical in protein structures (Luo et al., 2022).

In this study, the SPA was selected for feature wavelength extraction due to its efficiency in reducing multicollinearity and its low computational cost. While SPA has proven effective for selecting a compact subset of informative variables, alternative approaches such as Genetic Algorithms (Zhang et al., 2023), Random Forest (Shi et al., 2025)-based importance ranking, or Competitive Adaptive Reweighted Sampling (Huang et al., 2025) have also shown promise in hyperspectral feature selection tasks. These methods offer potential advantages in capturing global or nonlinear relationships but may require extensive parameter tuning or computational resources. Future research could investigate the comparative effectiveness of these approaches or explore hybrid strategies to enhance model performance and interpretability.

### 3.3 Analysis of modeling results

**Figure 5:**
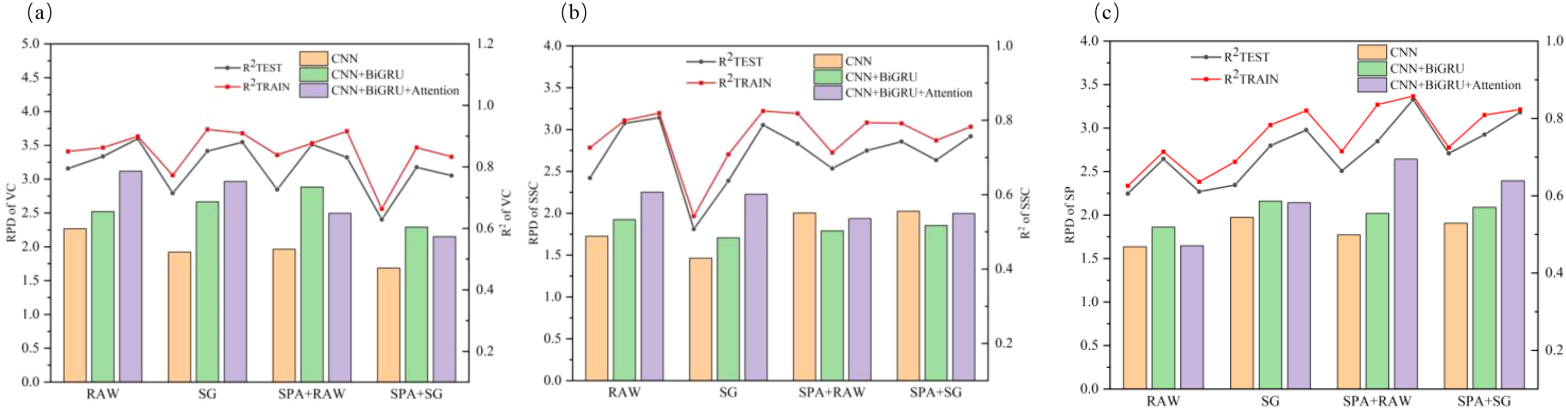
Comparative evaluation metrics of CNN, CNN-BiGRU, and CNN-BiGRU-Attention models with varied input modalities: **(a)** VC, **(b)** SSC, and **(c)** SP. SSC, soluble solids content; VC, ascorbic acid (vitamin C); SP, soluble protein.

A systematic evaluation was conducted to assess the predictive performance of CNN, CNN+BiGRU, and CNN+BiGRU+Attention across four spectral input configurations (RAW, SG, SPA + RAW, SPA + SG) for apple VC, SSC, and SP parameters, as comprehensively illustrated in **Figure 5**. The hybrid CNN+BiGRU+Attention architecture demonstrated superior modeling capabilities compared to the CNN alone. For vitamin C prediction, it achieved training and testing set R² values of 0.899 and 0.891, respectively, with RPD values consistently exceeding 3.100. In SSC quantification, reliable performance was maintained (testing set R² = 0.807, RPD = 2.337). Notably, the model attained enhanced prediction accuracy for soluble protein (testing set R² = 0.848, RPD = 2.673) through effective spectral feature integration. This performance enhancement was due to its multimodal feature extraction mechanism—the BiGRU establishes long-range contextual dependencies along forward-reverse wavelength dimensions, whereas the attention mechanism dynamically amplifies feature wavelengths critical to quality parameters through trainable weight matrices, ultimately forming biochemically interpretable feature representations (Zhao et al., 2025).

Distinct sensitivity patterns for spectral input configurations were observed among quality parameters. Optimal models for VC and SSC predictions utilized raw spectral inputs (RAW+CNN+BiGRU+Attention), confirming the autonomous feature extraction capability of deep networks through hierarchical nonlinear transformations (Pan et al., 2024). In contrast, SP prediction required SPA feature selection (SPA+RAW+CNN+BiGRU+Attention), wherein optimized input dimensionality facilitates focused modeling on characteristic absorption bands at 403, 430, 551, 617, and 846 nm to effectively mitigate noise interference in high-dimensional data, which is consistent with the findings of Xu et al (Xu et al., 2020). Raw spectral inputs systematically outperformed SG counterparts across all optimal models, contrasting conventional spectral analysis paradigms. This phenomenon suggests that deep neural networks preserve intricate non-linear correlations in raw data through adaptive feature learning, whereas excessive smoothing may disrupt dynamic coupling relationships between spectral responses and protein contents (Merzlyak et al., 2003).

### 3.4 External validation

**Figure 6:**
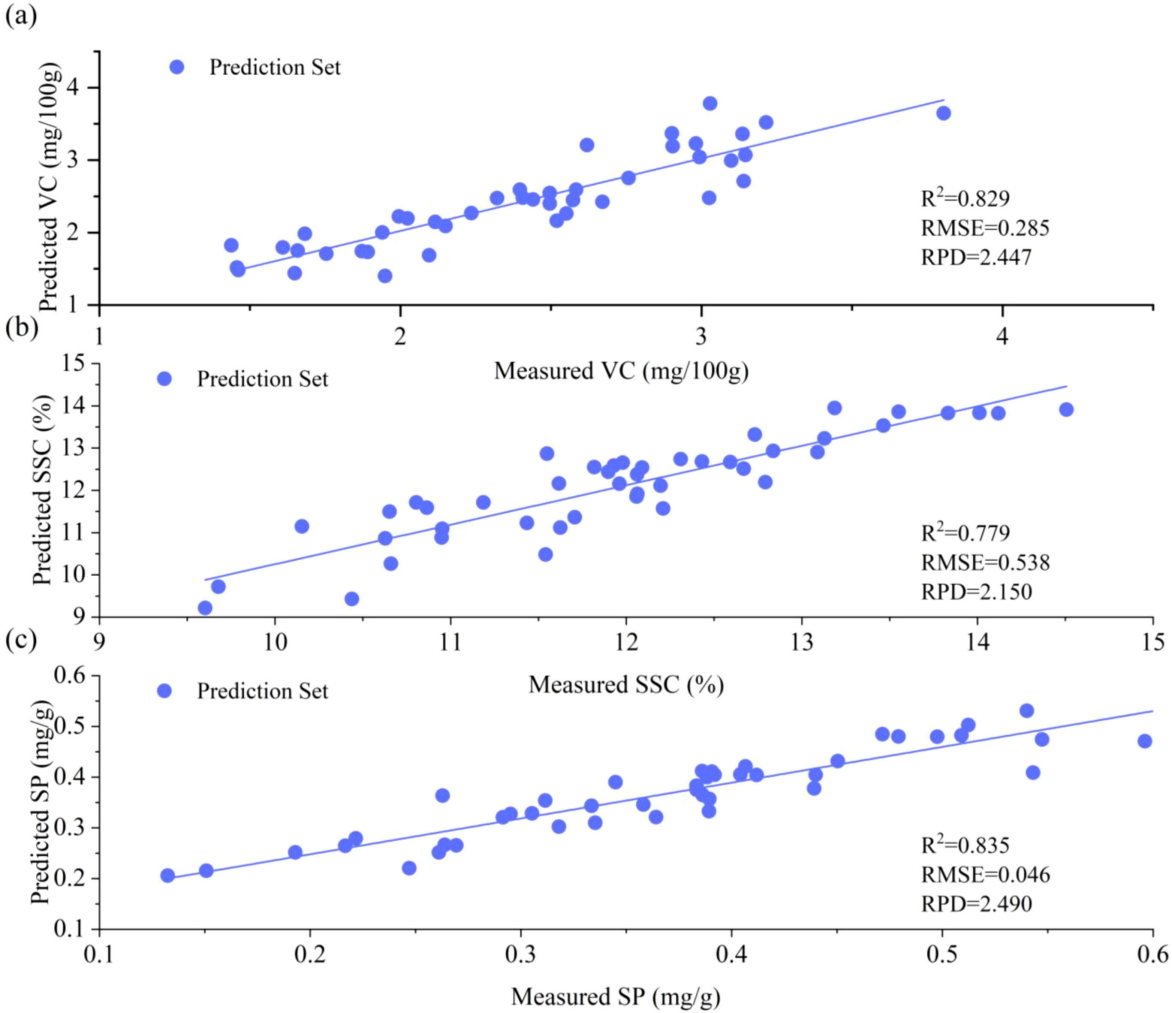
Results obtained with the external validation set: **(a)** VC, **(b)** SSC, and **(c)** SP. VC and SSC: RAW+CNN+BiGRU+Attention; SP: SPA+RAW+CNN+BiGRU+Attention.

To evaluate the generalization robustness of optimal models, a cross-year and cross-cultivar external validation protocol was implemented. The CNN+BiGRU+Attention architecture, initially trained on the 2023 dataset, was validated using an independent 2024 sample set (n = 48) comprising four cultivars (YT, GG, HN, and Red Fuji) and three geographical origins (Red Fuji apples from Shunping County, Hebei Province; Yantai, Shandong Province; and Aksu, Xinjiang Uygur Autonomous Region, n = 8 each). As shown in **Figure 6**, the external validation yielded robust performance metrics: VC (R²= 0.829, RPD = 2.447), SSC (R²= 0.779, RPD = 2.150), and SP (R²= 0.835, RPD = 2.490). Comparative analysis revealed minor reduction in performance compared to previous test set performance: VC exhibited a 6.95% reduction in *R²* and 21.49% reduction in RPD, SSC showed 3.46% R² and 8.00% RPD reductions, and SP demonstrated minimal degradation (1.53% R² and 5.75% RPD reductions). Notably, all the RPD values exceeded the benchmark threshold of 2.00 (**Figure 6**), confirming that the model retained stable spectral-component mapping relationships across different years and its cross-year industrial applicability.

The CNN-BiGRU-Attention model proposed in this study provides an innovative framework for non-destructive detection of apple quality. Future research can deepen its industrial applications from multiple dimensions. First, interpretable deep learning frameworks (e.g., SHAP and LIME) should be introduced to quantitatively analyze the contribution rates of different spectral bands to predict VC, SSC, and SP, and characteristic absorption peaks that are directly related to chemical components should be identified. This will enhance the transparency of model decisions and provide spectral band optimization guidance for portable sensor design. Second, multimodal data on apple growth environments should be integrated by combining spectral data with temperature, humidity, light, and soil nutrient parameters to establish “environment-quality” relationships, and align environmental temporal features with spectral responses through cross-modal attention mechanisms. Third, a lightweight apple quality grading system should be developed by compressing model scale through neural network pruning and quantization-aware training techniques.

## 4 Conclusions

This study established a CNN-BiGRU-Attention deep learning framework based on Vis-NIR spectroscopy to achieve high-accuracy prediction of apple quality parameters (VC, SSC, and SP) across six cultivars and geographical origins. By synergistically integrating the local feature extraction capability of CNN, long-range wavelength dependency modeling via BiGRU, and dynamic enhancement of critical spectral regions through attention mechanisms, the model demonstrated exceptional performance with raw spectral inputs. Optimal full-spectrum predictions were achieved for VC and SSC, but SP quantification required supplementary SPA to focus on chemically informative bands (403, 430, 551, 617, and 846 nm). A comparative analysis revealed that direct modeling of raw spectra significantly improved non-linear pattern recognition efficiency compared to that of SG data, suggesting the autonomous feature optimization capacity of deep architectures.

Cross-year external validation using an independent 2024 dataset (n = 48) confirmed model robustness, with VC, SSC, and SP achieving R² values of 0.829, 0.779, and 0.835, respectively, while maintaining RPD values consistently above 2.0. These results validate the reliability of spectral-component mapping across temporal and spatial variations. The proposed framework provides a scalable deep learning solution for non-destructive fruit quality evaluation, demonstrating significant potential for industrial applications in multi-cultivar and cross-regional scenarios. Future studies should expand the sample size and incorporate additional cultivars to further enhance prediction robustness across diverse agricultural conditions.

## Data Availability

The raw data supporting the conclusions of this article will be made available by the authors, without undue reservation.
